# Supplementary material for: Drug repurposing for aging research using model organisms
Source: Aging Cell. 2017 Jun 16;16(5):1006–15. doi: 10.1111/acel.12626 (PMC5595691; doi:10.1111/acel.12626)
Supplement: Supplementary file 7 — Data S1 Zip‐Archive of all report cards. [file ACEL-16-1006-s007.zip › RC_LDP.pdf]

# LDP

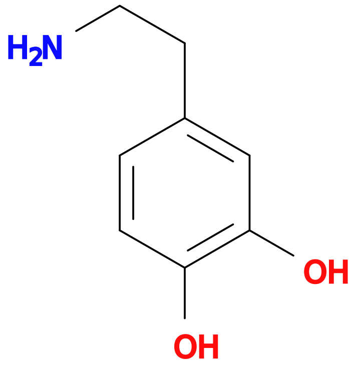


**Database identifiers**

| | |
|---|---|
| ChEMBLCompound | CHEMBL59 |
| DrugBank | DB00988 |
| CHEBI | 18243 |
| eMolecules | 739600 |

## Ranking

| | Rank | Score |
|---|---|---|
| Drosophila | 392/697 | 0.37 |
| C. elegans | 255/591 | 0.216 |

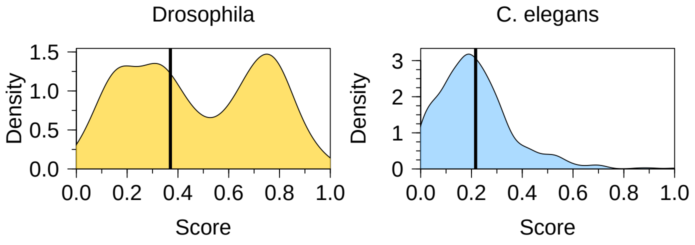


| | Ageing implication | Domain conservation | Binding site conservation | Binding affinity | Bioavailability | Lipinski | Promiscuity | Purchasability | Drug approval | **Total** |
|---|---|---|---|---|---|---|---|---|---|---|
| Drosophila | 1.0 | 0.922 | 0.812 | 0.253 | (0.9) | 0.0 | -0.0 | 0.1 | 0.1 | 0.37 |
| C. elegans | 1.0 | 0.748 | 0.366 | 0.253 | 0.244 | 0.0 | -0.0 | 0.1 | 0.1 | 0.216 |

## Names

- **dopamine**
- 2-(3,4-dihydroxyphenyl)ethylamine
- 3,4-Dihydroxyphenethylamine
- 4-(2-Aminoethyl)-1,2-benzenediol
- 4-(2-Aminoethyl)benzene-1,2-diol
- 4-(2-aminoethyl)catechol
- 4-(2-aminoethyl)pyrocatechol
- Carbilev
- Deoxyepinephrine
- Hydroxytyramin
- Intropin
- Parcopa

· Sinemet

## Roles

cardiotonic drug, beta-adrenergic agonist, dopaminergic agent, sympathomimetic agent, human metabolite

## Status

| | |
|---|---|
| Approved drug (according to ChEMBL) | Yes |
| Administration Route | Intravenous drip |
| Number of Rule of 5 violations | 0 |
| Binding affinity to original target in log units (RF-Score prediction) | 3.92 |
| Burns *C. elegans* bioavailability prediction | -2.42 |

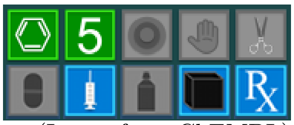

(Image from ChEMBL)

## Medical Information

**Indicatation:** For the correction of hemodynamic imbalances present in the shock syndrome due to myocardial infarction, trauma, endotoxic septicemia, open-heart surgery, renal failure, and chronic cardiac decompensation as in congestive failure
**Mechanism of action:** Dopamine is a precursor to norepinephrine in noradrenergic nerves and is also a neurotransmitter in certain areas of the central nervous system. Dopamine produces positive chronotropic and inotropic effects on the myocardium, resulting in increased heart rate and cardiac contractility. This is accomplished directly by exerting an agonist action on beta-adrenoceptors and indirectly by causing release of norepinephrine from storage sites in sympathetic nerve endings. In the brain, dopamine actas as an agonist to the five dopamine receptor subtypes (D!, D2, D3, D4, D5).
**Toxicity:** LD50 oral mice = 1460 mg/kg, LD50 oral rats = 1780 mg/kg. Spasm or closing of eyelids, nausea, vomiting, cardiac arrhythmias, involuntary movements of the body including the face, tongue, arms, hand, head, and upper body; hypotension, haemolytic anaemia, urinary retention, duodenal ulcer, sialorrhea, ataxia, abdominal pain, dry mouth, nightmares, tachypnoea, bruxism, confusion, and insomnia.
**Metabolism:** Biotransformation of dopamine proceeds rapidly to yield the principal excretion products, 3-4-dihydroxy-phenylacetic acid (DOPAC) and 3-methoxy-4-hydroxy-phenylacetic acid (homovanillic acid, HVA).
**Retail:** American Regent, B. Braun Melsungen AG, Baxter International Inc., Bristol-Myers Squibb Co., Cardinal Health, General Injectables and Vaccines Inc. and 4 more suppliers
Dopamine 400 mg-d5w 250 ml - 0.04 USD / ml
Dopamine 800 mg-d5w 500 ml - 0.05 USD / ml
Dopamine 40 mg/ml vial - 0.15 USD / ml

(Information from DrugBank)

## Compound Target Characteristics

### Superoxide dismutase [Cu-Zn]

Best gene implication in ageing for this target family came from gene P61851 annotated in UniProt release 2014_02. Annotation GO 8340 (determination of adult lifespan) was Inferred from Mutant Phenotype

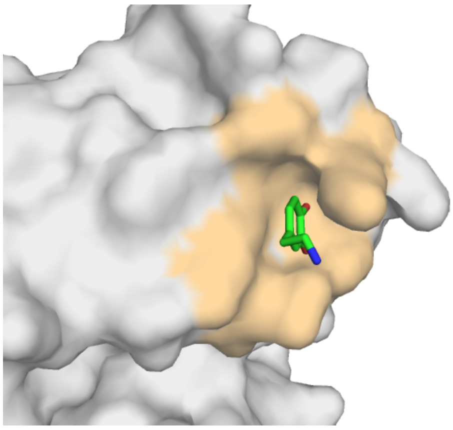

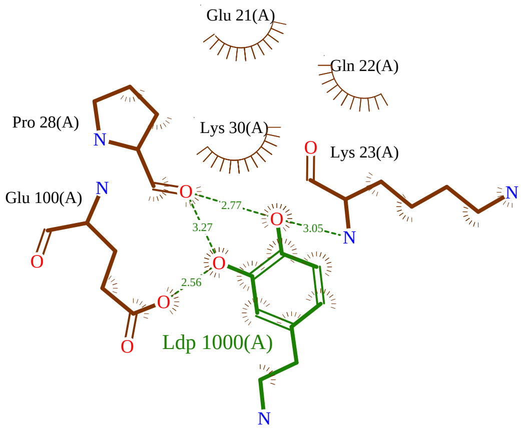


| protein | amino acids contacts (binding site) | | | | | |
|---|---|---|---|---|---|---|
| PDB:4a7v:chainA:P00441 | E | Q | K | P | K | E |
| sp:P00441:SODC_HUMAN | E | Q | K | P | K | E |
| sp:P07632:SODC_RAT | E | Q | K | P | V | E |
| tr:Q6LDS4:Q6LDS4_RAT | E | Q | K | P | V | E |
| sp:P08228:SODC_MOUSE | E | Q | K | P | V | E |
| sp:P61851:SODC_DROME | E | Q | E | P | K | T |
| tr:G5ECR5:G5ECR5_CAEEL | D | Q | - | F | K | S |
| sp:P34461:SODE_CAEEL | D | Q | - | F | K | S |
| sp:P00445:SODC_YEAST | E | Q | A | P | T | K |

| | whole protein | | domain-based | | contact-based | |
|---|---|---|---|---|---|---|
| protein | ident | simil | ident | simil | ident | simil |
| PDB:4a7v:chainA:P00441 | 0.99 | 1.0 | 0.99 | 1.0 | 1.0 | 1.0 |
| sp:P00441:SODC_HUMAN | 1.0 | 1.0 | 1.0 | 1.0 | 1.0 | 1.0 |
| sp:P07632:SODC_RAT | 0.83 | 0.94 | 0.83 | 0.94 | 0.83 | 0.85 |
| tr:Q6LDS4:Q6LDS4_RAT | 0.82 | 0.93 | 0.82 | 0.93 | 0.83 | 0.85 |
| sp:P08228:SODC_MOUSE | 0.84 | 0.94 | 0.83 | 0.94 | 0.83 | 0.85 |
| sp:P61851:SODC_DROME | 0.61 | 0.84 | 0.6 | 0.85 | 0.67 | 0.81 |
| tr:G5ECR5:G5ECR5_CAEEL | 0.35 | 0.57 | 0.44 | 0.71 | 0.33 | 0.37 |
| sp:P34461:SODE_CAEEL | 0.35 | 0.57 | 0.44 | 0.71 | 0.33 | 0.37 |
| sp:P00445:SODC_YEAST | 0.54 | 0.83 | 0.55 | 0.84 | 0.5 | 0.63 |

## Sod (FBgn0003462) associated phenotypes

calorie restriction conditional, chemical conditional, chemical sensitive, heat sensitive, increased cell number, locomotor behavior defective, long lived, male limited, oxidative stress response defective, partially, partially lethal - majority die, progressive, radiation sensitive, reduced, short lived

(Information from FlyBase)

## Sod (UniProt:P61851) annotation

**Function**: Destroys radicals which are normally produced within the cells and which are toxic to biological systems.
**Cofactor**:Cu cationNote=Binds 1 copper ion per subunit.;
**Cofactor**: Zn(2+)Note=Binds 1 zinc ion per subunit.;
**Subunit**: Homodimer.
**Subcellular location**: Cytoplasm.

(Information from UniProt)

## sod-4 (UniProt:G5ECR5) annotation

**Function**: Destroys radicals which are normally produced within the cells and which are toxic to biological systems. (RuleBase:RU000393).
**Cofactor**: Cu cation Evidence=(RuleBase:RU000393); Note=Binds 1 copper ion per subunit. (RuleBase:RU000393);

**Cofactor**: Zn(2+) Evidence=(RuleBase:RU000393); Note=Binds 1 zinc ion per subunit. (RuleBase:RU000393);

(Information from UniProt)

**sod-4 (UniProt:P34461) annotation**

**Function**: Destroys radicals which are normally produced within the cells and which are toxic to biological systems.
**Cofactor**:Cu cationNote=Binds 1 copper ion per subunit. ;
**Cofactor**: Zn(2+)Note=Binds 1 zinc ion per subunit. ;
**Subcellular location**: Isoform 1: Secreted, extracellular space.
**Subcellular location**: Isoform 2: Membrane ; Peripheral membrane protein
**Tissue specificity**: Isoform 2 is preferentially expressed in eggs.

(Information from UniProt)
